# Perifoveal Exudative Vascular Anomalous Complex (PEVAC): Retinal Vascular Density Findings

**DOI:** 10.3390/jcm13226879

**Published:** 2024-11-15

**Authors:** Hamzah Aweidah, Deborah Cosette, Natan Lishinsky-Fischer, Tarek B. Eshak, Tomer Batash, Itay Chowers, Tareq Jaouni, Nadav Levinger, Jaime Levy

**Affiliations:** 1 Department of Ophthalmology, Hadassah Medical Center, Faculty of Medicine, The Hebrew University of Jerusalem, Jerusalem 91120, Israel; hamzah.aweidah@mail.huji.ac.il (H.A.); natan.lishinsky@mail.huji.ac.il (N.L.-F.); batasht@gmail.com (T.B.); chowers@hadassah.org.il (I.C.); tareq@hadassah.org.il (T.J.); nadav.levinger@gmail.com (N.L.); 2 Carl Zeiss Meditec, Inc., Dublin, CA 94568, USA; deborah.cosette@zeiss.com; 3 Department of Public Health Sciences, College of Health Professions, Slippery Rock University, Slippery Rock, PA 16057, USA; tarek.eshak@sru.edu

**Keywords:** PEVAC, OCTA, vessel density, ARI network

## Abstract

**Objectives**: This study aimed to describe the clinical, optical coherence tomography (OCT) and OCT angiography (OCTA) findings and characteristics in patients with perifoveal exudative vascular anomalous complex (PEVAC) and compare the macular vascular density with the age-matched control group. **Methods**: We conducted a case–control study to compare demographic information, clinical observations, and OCT/OCTA findings in eyes with PEVAC (*n* = 5 eyes in 5 patients) and a control group of subjects matched for age (*n* = 9). The Advanced Retina Imaging (ARI) network algorithms were utilized to evaluate OCTA observations. Statistical analysis was performed by the nonparametric Mann–Whitney U test. **Results**: Patients with PEVAC had a mean (±SD) age at presentation of 70 ± 12.6 years, the mean follow-up period was 7.8 ± 5.2 months, and unilateral disease was observed. Four out of the five patients in our cohort had a history of systemically treated hypertension and dyslipidemia. Three eyes had lesions in the inner temporal retinal zone, while the remaining two eyes had lesions in the inner inferior or central zone. Retina slab analysis using OCTA showed no significant difference in vascular density parameters between the PEVAC and control groups. **Conclusions**: Although limited by a small sample size, our study suggests that macular vessel density shows no significant difference between PEVAC cases and control eyes.

## 1. Introduction

Previous research reports that perifoveal exudative vascular anomalous complex (PEVAC) consists of a large, isolated, well-defined perifoveal aneurysmal abnormality that occurs in otherwise healthy individuals with no signs of systemic or local vasculopathy, as well as in those with no signs of age-related macular degeneration (AMD) or microvascular risk factors such as diabetes mellites, systemic hypertension, and blood dyscrasia [1].

Macular telangiectasia (MacTel) type 1 can resemble PEVAC lesions; however, it is characterized by unilateral aneurysmal dilatations that may extend beyond the macula to include the temporal macular vasculature. This condition is often accompanied by adjacent cystoid macular edema (CME) and exudates, as well as noticeable peripheral vascular changes. It primarily affects males around the age of 40 [2,3].

Multimodal retinal imaging methods such as optical coherence tomography angiography (OCTA) were applied to study the pathophysiological mechanisms underlying PEVAC [4,5,6,7,8,9,10,11,12,13,14]; however, little is currently known regarding its pathogenesis. Based on OCTA, the term nonexudative perifoveal vascular anomalous complex (nePVAC) was first proposed by Sacconi et al. to describe an aneurysmal lesion with a detectable internal flow that changes during subsequent follow-up visits and disappears when the aneurysm has resolved. Increased flow in the aneurysmal lesion indicates the development of exudative complications and the progression of the lesion from the subclinical pre-exudative stage (nePVAC) to the exudative stage (ePVAC) [7]. The flow signal of the aneurysmal lesion and its connection with the superficial capillary plexus (SCP) and/or deep capillary plexus (DCP) in the absence of any other vascular anomalies or signs of anastomosis between the retinal capillary plexuses and the choriocapillaris was also described [4,5,7,8,9,14].

Microvascular quantification measurements are a key parameter derived from OCTA images. One of these measurements is vessel density, which has been utilized for the quantitative assessment of ocular vascular structures in diabetic retinopathy [15], retinal vein occlusion [16], choroidal neovascularization [17,18], and inherited retinal dystrophies [19]. Microvascular quantification measurements can provide important insights into the activity and pathogenesis of PEVAC lesions, but this has not been described before. Therefore, the aim of this study is to characterize the microvascular circulation in PEVAC using OCTA and compare it to an age-matched control group.

Here, we examined the clinical features of PEVAC and investigated its pathogenesis, describing the underlying microvasculature measured using swept-source OCTA (SS-OCTA) images combined with the web-based Advanced Retina Imaging (ARI) network platform and comparing the results with control eyes.

## 2. Materials and Methods

### 2.1. Study Design and Population

**Figure 1:**
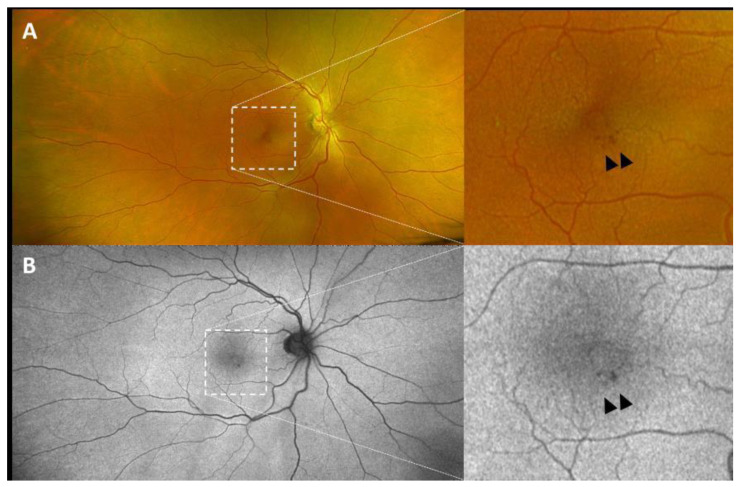
Pseudocolor (**A**) and autofluorescence (**B**) ultra-widefield Optos images showing a perifoveal isolated aneurysmal lesion of P-5 (arrowheads).

This was a retrospective, single-center case–control analysis comparing the macular vessel density between patients with PEVAC and a control group presented at Hadassah Medical Center in Jerusalem, Israel, from March 2018 through July 2021. The diagnosis of PEVAC was based on fundus examination, SD-OCT, and OCTA images. PEVAC lesions were identified as isolated, round hyperreflective walls surrounding vessel lumens, located in the superficial and/or deep retinal layers of the perifoveal region. Patients with PEVAC were included in the study if they did not exhibit any clinical signs of diabetic retinopathy, hypertensive retinopathy, retinal vascular occlusion, or myopic degenerative retinal disease (Figure 1).

Patients with PEVAC were excluded from the analysis if they either lacked OCTA scans using PLEX Elite 9000 (Carl Zeiss Meditec, Inc., Dublin, CA, USA); if the OCTA acquisition did not cover the 6 × 6 mm area around the fovea; or if they presented with any additional retinal or choroidal vascular abnormalities, previous medical or surgical retinal treatment, diabetic retinopathy, uncontrolled systemic hypertension, significant ocular media opacity, and/or had a follow-up duration of less than three months.

To increase our clinical and OCTA-based understanding of PEVAC, we included an age-matched control group from the same time period. Subjects in the control group had no ocular complaints at the time of examination and exhibited no clinical signs of diabetic retinopathy, hypertensive retinopathy, retinal vascular occlusion, or myopic degenerative retinal disease. They also had no history of ocular surgery (except for cataract removal >1 year before inclusion in the study) and showed no other signs of retinal or choroidal disease. One subject from the control group was randomly selected for each patient with PEVAC.

The study was conducted in accordance with the tenets established by the Declaration of Helsinki for research involving human subjects and was approved by the Institutional Review Board of the Hadassah Medical Center (IRB approval number: HMO 20-0302). All subjects provided written informed consent for their clinical data to be used for research purposes.

### 2.2. Image Acquisition, Validation, and Analysis

OCTA examinations were conducted on all patients included in the analysis and on control subjects using an Angio 6 × 6 mm scan pattern centered on the fovea. The scans were performed with a SS-OCTA device PLEX Elite 9000 (Carl Zeiss Meditec, Inc., Dublin, CA, USA), based on the Optical Microangiography (OMAG) algorithm [20]. The device runs with 100,000 A-scans per second with a central wavelength of 1060 nm (full bandwidth: 1000–1100 nm, measured in nanometers).

To reduce motion-related artifacts, all images were acquired using a tracking system: FastTrac™ (Carl Zeiss Meditec, Inc., Dublin, CA, USA), the inbuilt retinal-tracking technology. Furthermore, each study subject underwent two separate rounds of Angio 6 × 6 mm OCTA imaging for flow detection, and the volumes were averaged.

**Figure 2:**
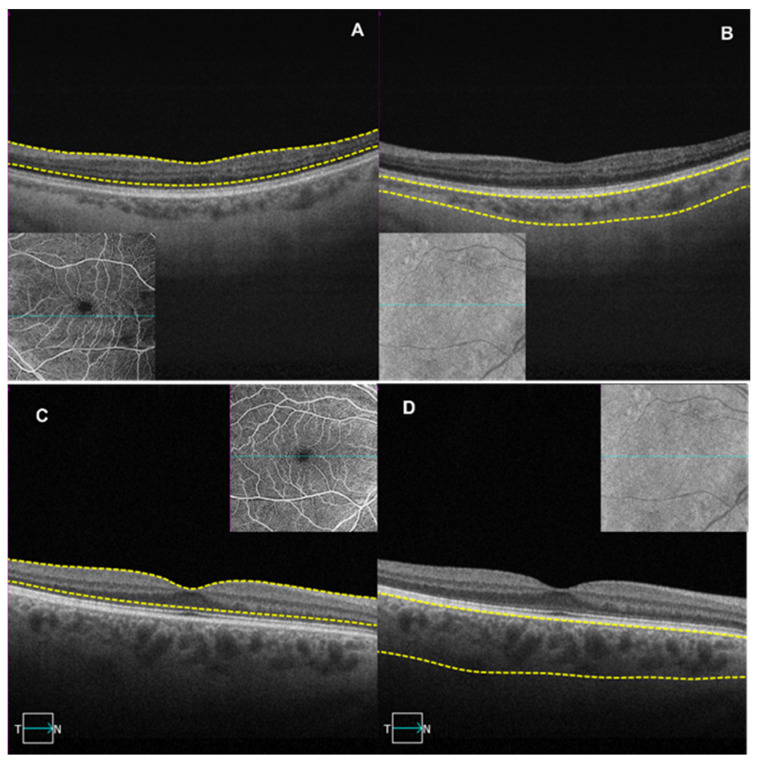
B-scan with slab segmentation (highlighted between the yellow dotted lines) and OCTA imaging PEVAC eye and a control eye. Panel (**A**) represents the retina slab of the left eye in patient P-2. Panel (**B**) represents the choroid slab of the left eye in patient P-2. Panel (**C**) represents the retina slab of the right eye in patient C-3. Panel (**D**) represents the choroid slab of the right eye in patient C-3.

After obtaining the OCTA images, two retina specialists (authors J.L. and T.J.) thoroughly examined them to verify the accuracy of the upper and lower boundaries defined as the inner limiting membrane (ILM) and retinal pigment epithelium (RPE), respectively. In all cases, automated segmentation was found to be accurate; thus, manual correction was not required (Figure 2). The vascular beds were defined using automated segmentation, resulting in distinct layers of the retina known as ‘retina slabs’, each based on three depths. Specifically, the superficial capillary plexus (SCP) extends from the ILM to the inner plexiform layer (IPL), while the deep capillary plexus (DCP) extends from the IPL to the outer plexiform layer (OPL). The device’s projections removal algorithm was applied to DCP images.

Any identifiable information was removed from the scans. Poor-quality images were excluded from the analysis, defined as having a signal strength index < 8 or images with either significant motion-related artifacts or extensive incorrect segmentation.

For the PEVAC group, we included the OCTA images acquired at the time of diagnosis. OCTA images were analyzed using several prototype algorithms provided by the manufacturer (Carl Zeiss Meditec, Inc., Dublin, CA, USA) on the ARI network.

The Advanced Research Imaging (ARI) network is a cloud-based platform from ZEISS (Carl Zeiss Meditec AG, Jena, Germany) designed to support clinical research. Following the upload of the anonymized data (OCT-OCTA images from PLEX Elite 9000 only) on this portal, data processing with Zeiss proprietary prototype algorithms (D = data visualization, image quality, or quantification analysis for instance) can be used.

In this study, we used the “macular density” version 0.7.3 algorithm for analysis. Automated layer segmentation is provided with multilayer segmentation (segmentation of 8 layers), and projection artifacts are resolved. It provides perfusion density measurement, vessel density measurement, and FAZ measurement for SCP, DCP, or full retina slabs.

**Figure 3:**
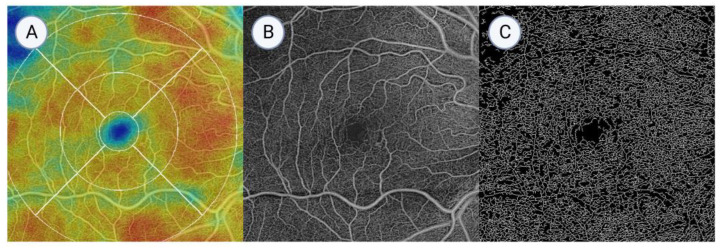
Optical coherence tomography angiography (OCTA) imaging of the right eye in patient P-2. (**A**) Superficial angio en face perfusion map showing the 9 zones (the central zone and the inner and outer nasal, temporal, superior, and inferior zones). (**B**) A retina slab image of the same eye shown in (**A**). (**C**) A skeletonized image of the retina slab shown in (**B**).

Processing was performed on the overall volume, and the results were collected within the ETDRS grid (6 mm) and its nine zones (i.e., the central zone and the inner and outer nasal, temporal, superior, and inferior zones); see Figure 3 and Supplementary Table S1.

Perfusion density was defined as the total perfused area divided by the total image area, based on a binary image. Vessel density, on the other hand, was defined as the total vessel length (in mm) divided by the total image area (in mm^2^). This calculation was based on a skeletonized image, where vessel diameter did not affect the measurements, and all vessels were treated equally [21,22].

In this specific study, since the PEVAC lesions were not localized to a single SCP or DCP slab, we used the retina slab for both the PEVAC and control groups to analyze the macula (ETDRS grid sectors).

### 2.3. Statistical Analysis

Statistical analysis was performed using Prism (version 9, GraphPad, San Diego, CA, USA). The primary outcome was SS-OCT metrics. Continuous non-normally distributed variables were analyzed by the nonparametric two-tailed Mann–Whitney U test to compare the ranks between the two groups. Significance was determined for differences with a *p*-value of <0.05.

## 3. Results

During the study period, a total of nine patients were diagnosed with PEVAC, of whom five eyes (one right eye and four left eyes) from five Caucasian patients (three male and two female) were eligible for analysis. Four patients were excluded because they did not meet the inclusion criteria, either due to not having two separate rounds of Angio 6 × 6 OCTA imaging performed using the PLEX Elite device, or because their measured macular vascular density area differed from the required 6 × 6 mm scan area included in the analysis. The mean (±SD) age of these five patients at the time of PEVAC diagnosis was 70 ± 12.6 years (median: 73 years; range: 56–85 years). Table 1 summarizes the baseline characteristics of these five patients. Four patients had medically controlled systemic hypertension and dyslipidemia, and one patient had medically controlled non-insulin-dependent diabetes mellitus. None of the patients had diabetic retinopathy, hypertensive retinopathy, retinal vascular occlusion, myopic degenerative retinal disease, previous ocular surgery (other than cataract removal performed >1 year before baseline), or any other retinal or choroidal disease in the affected eye(s). The mean follow-up period for the patients was 7.8 ± 5.2 months (median: 4 months; range: 4–14 months).

The control group included nine eyes (six right eyes and three left eyes) in nine Caucasian subjects (five male and our female). The mean age of the control group was 66.6 ± 7.1 years (median: 63 years; range: 59–78 years). One subject had systemic hypertension and non-insulin-dependent diabetes mellitus controlled by medications, and another had hypertension and dyslipidemia, while two others had medically controlled hypertension. The remaining subjects did not have any systemic illnesses. Two of the control eyes had previously undergone uneventful cataract surgery >1 year before the study (Table 1).

We found no significant difference between groups with respect to age, gender, hypertension, and DM type 2 (*p* = 0.84, 1.00, 0.30, and 1.00). Subjects with PEVAC had significantly more cases of dyslipidemia (*p* = 0.02).

At the time of PEVAC diagnosis, all except one patient were presented with reduced visual acuity in the affected eye. The mean BCVA in the affected eyes was 20/30 ETDRS equivalent, corresponding to 0.14 ± 0.05 logMAR (logarithm of the minimum angle of resolution) units (median: 0.1 logMAR; range: 0.1–0.2 logMAR), and the mean central macular thickness (CMT) was 321.4 ± 67 μm (median: 286 μm; range: 271–433 μm) (Table 2).

**Figure 4:**
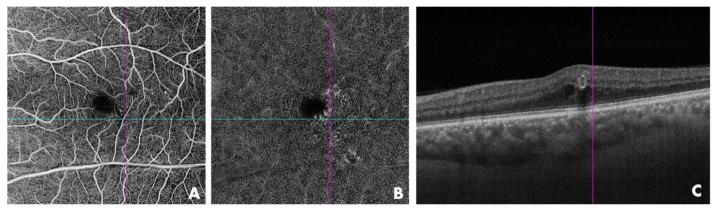
OCTA of the affected eye in patient P-4, confirming the presence of perifoveal capillary abnormalities (crossing lines) in the superficial capillary plexus (**A**) and deep capillary plexus (**B**). (**C**) B-scan image showing the presence of an isolated, well-defined perifoveal aneurismal lesion (purple line) with small intraretinal cystoid macular edema close to the PEVAC lesion.

In four out of the five eyes, the PEVAC lesions were surrounded by intraretinal cystic spaces, whereas none of the affected eyes showed any evidence of subretinal fluid (Figure 4). All five eyes had a normal vitreoretinal interface and ellipsoid zone structure. Following PEVAC diagnosis, one eye was treated with antivascular endothelial growth factor (anti-VEGF) injections, specifically, the left eye in patient P-1, which received a total of 10 injections (Table 2).

The mean BCVA in the control eyes was equivalent to 20/23 ETDRS, corresponding to 0.06 ± 0.08 logMAR (median: 0.05 logMAR; range: 0.0–0.22 logMAR). SD-OCT data were not obtained as part of the evaluation for the control group.

**Figure 5:**
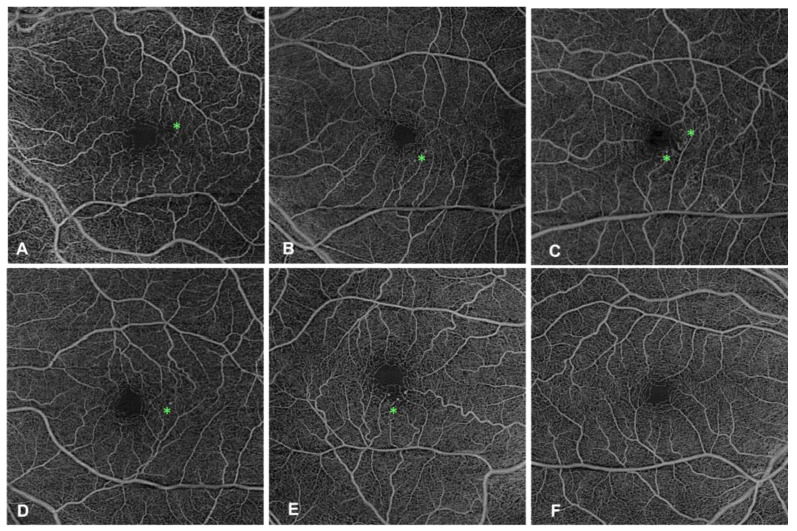
OCTA images of the retina slab in the affected eye of PEVAC patients and one control eye. Panel (**A**) represents the left eye of patient P-1, panel (**B**) shows the left eye of patient P-2, panel (**C**) displays the left eye of patient P-3, panel (**D**) shows the left eye of patient P-4, panel (**E**) shows the right eye of patient P-5, and panel (**F**) shows the right eye of control subject C3. The PECAC lesion(s) are marked with a green asterisk.

Next, we compared vessel density in the retina slab between the PEVAC group and the control group for the inner and outer nasal, temporal, superior, and inferior retinal zones (Figure 5). Based on our sample size, we performed the nonparametric Mann–Whitney U test to assess the differences between the two groups. This test does not assume normality on the data and thus makes it an appropriate choice for our analysis. The results of the Mann–Whitney U test did not reveal any statistically significant difference between the PEVAC and control groups in any of the zones (Supplementary Table S2).

Subsequently, we analyzed the effect of PEVAC lesions on the macular vascular density angio en face in the superficial capillary plexus and deep vascular plexus. Among the five affected eyes, the PEVAC lesions occurred in the inner temporal zone in three eyes, while in the remaining two eyes, the lesions occurred in the inner inferior zone (*n* = 1), or central zone (*n* = 1). However, this difference was not significant, and because the PEVAC lesions were not localized to a single SCP or DCP slab, we used the retina slab for the macular vascular density analysis (Supplementary Table S2).

## 4. Discussion

Here, we describe the clinical, SD-OCT, and SS-OCT findings of five eyes in five patients with PEVAC. All except one patient with PEVAC presented with reduced visual acuity associated with metamorphopsia. Importantly, four out of the five affected eyes presented with intraretinal cystic spaces surrounding the area of the PEVAC lesions. Using the ARI network, we quantified vessel density on OCTA images and compared the results with an age-matched control group. No statistically significant differences were found in the different retina slabs between PEVAC cases and control eyes.

The neurosensory retina primarily receives blood from the retinal circulation, whereas the choroidal vasculature supplies the outer retina, including the photoreceptors and retinal pigment epithelium (RPE). The fovea exclusively receives its blood supply from the choroidal vasculature, forming the foveal avascular zone (FAZ) with disease-specific topographic patterns [23]. The retinal circulation presents two primary types of aneurysms: retinal arterial macroaneurysms (large and involving first-order arterial branches) [24,25] and microaneurysms (smaller dilations of retinal capillaries) [26]. Arterial macroaneurysms may occur independently, often in the elderly and possibly associated with systemic hypertension, or in complex conditions such as idiopathic retinal vasculitis, aneurysms and neuroretinitis (IRVAN) syndrome, Coats’ disease, and Eales’ disease [27,28,29]. Conversely, microaneurysms are commonly observed in eyes with diabetic retinopathy, hypertensive retinopathy, vascular remodeling following retinal vein occlusions, and other specific conditions affecting the retinal vasculature (e.g., MacTel type 1, MacTel type 2 and adult Coats disease) [30,31]. Recently, aneurysmal dilations of the DCP have also been identified as part of the spectrum of vascular changes seen in AMD type 3, including its proliferative and pre-proliferative stages (deep retinal age-related microvascular anomalies, DRAMA) [32]. It is also known that isolated microaneurysms of retinal vessels can occur in healthy individuals without signs of diabetic retinopathy or other vascular disorders [33]. Therefore, some authors propose that aneurysmal dilations of retinal vessels located in the perifoveal region, not attributable to any known retinal pathology, should be termed “PEVAC” and categorized accordingly. Attempting to classify all perifoveal microaneurysms under a single definition poses inherent risks, as entities that are fundamentally different may be incorrectly grouped together. Nevertheless, even if every isolated perifoveal microaneurysm were labeled as PEVAC, the diagnosis must be made through exclusion.

The use of OCTA in analyzing various retinal vascular diseases allows for the segmentation and separation of specific retinal vascular plexuses, providing a three-dimensional visualization of the retinal microvasculature and the choriocapillaris. This allows for the detection of blood flow within the vasculature and the ability to analyze volumetric retinal microvascular networks using various algorithms [34]. One such algorithm is the macular density algorithm, which can be used to visualize and quantify the FAZ, as well as to visualize and calculate vascular density in the surrounding tissue [35].

Thanks to their ability to both quantitatively and qualitatively assess the retinal vasculature, these imaging modalities have begun to transform our understanding of the molecular pathogenesis of retinal diseases and play an increasing role in the early diagnosis and management of various retinal abnormalities. PEVAC lesions were previously described as hyperreflective on OCTA, often associated with a reflective wall and a dark lumen. Moreover, the flow signal is correlated with the aneurysmal lesion directly connected to the SCP and/or DCP, while the presence of multiple lesions in the same eye can show as multiple flow signals with no other associated vascular anomalies or signs of anastomosis between the retinal capillary plexuses and choriocapillaris [4,5,7,8,9,14]. Additionally, the term “microangiopathy” has also been used to describe PEVAC lesions based on the previous description that these lesions can regress—and even disappear—over time without any intervention [8,36]. In our small cohort consisting of five patients, none of the PEVAC lesions regressed in any of the affected eyes.

Although the pathogenesis of aneurysmal microangiopathy in PEVAC lesions is poorly understood, it is believed to involve a complex interplay of various factors, including changes in the retinal vasculature with focal endothelial cell injury related to non-perfusion and/or neovascularization [4]. Pericytes and endothelial cells are the two principal cell types in the retinal vasculature, which share the same basement membrane and are critical for maintaining the integrity of the blood–retina barrier. Loss of pericytes can disrupt cell-to-cell communication, leading to aneurysm formation, edema, and ischemia, resulting in reduced wall strength and increased wall tension [37]. The breakdown of the basement membrane due to loss of pericytes has been described in aneurysmal microangiopathies and capillary rarefaction in diabetic retinopathy and PEVAC lesions. This can cause reduced wall strength and increased wall tension [36,38]. A possible explanation for this is the theory postulated by Spaide and Barquet [39], namely that these types of lesions may be the result of an aneurysmal expansion, possibly due to a weakening of the lesion wall caused by a defect in matrix metalloproteinases and an increase in wall tension.

In our PEVAC group, we found that four out of the five patients (80%) had medically treated primary hypertension with no ocular signs of systemic hypertension, compared to four out of the nine control subjects (44.4%). Furthermore, four out of the five PEVAC patients also suffered from dyslipidemia compared to one control subject. This finding may call into question the definition of PEVAC as a primary idiopathic lesion unrelated to the presence of any underlying systemic/retinal disease. A recent study comparing serum apolipoprotein B with retinal neurovascular structural alterations in patients with type 2 diabetes using OCTA found that individuals with dyslipidemia had significantly less vessel density and perfusion density than those with diabetes. This finding suggests that dyslipidemia may have a greater impact on retinal vascular health compared to diabetes [40]. Therefore, regular monitoring and management of hypertension and dyslipidemia may be crucial in preserving retinal vascular health. Additionally, these findings suggest that the definition of PEVAC as a primary idiopathic lesion unrelated to the presence of any underlying systemic/retinal disease may need to be reevaluated.

Since 2011, there has been a growing body of literature describing the clinical characteristics and the pathogenesis of PEVAC, including studies that have reported the presence of controlled systemic hypertension in a significant proportion of patients with this condition. For example, Sacconi et al. used multimodal imaging, including OCTA, to examine 15 patients with PEVAC and reported that 7 of these patients had controlled systemic hypertension [4]. In a later study designed to describe the pre-exudative stage of PEVAC, the same group reported that two of their six patients with PEVAC had systemic hypertension [7]. In addition, Kim et al. described the clinical characteristics of PEVAC in eight Korean patients and reported that five of these patients had controlled systemic hypertension [6]. Recently, Verhoekx et al. examined anatomical changes over time in a group of 21 patients with PEVAC, 11 of whom had controlled systemic hypertension [8]. Moreover, Smid et al. used imaging to compare the features of PEVAC and PEVAC-like lesions and found that 7 out of 10 patients with PEVAC had medically controlled hypertension [36]. PEVAC was also described in a case report of a highly myopic patient who also had medically controlled hypertension [41] and in a patient who presented with PEVAC and lamellar hole-associated epiretinal proliferation [12]. Based on these studies, PEVAC was described in 61 different patients, 33 of them documented that they were suffering from systemic hypertension. Furthermore, none of these studies have reported microvascular quantification measurements using OCTA, such as vessel density.

Hypertension can have a negative impact on various organs and organ systems. In the heart, it can affect the cardiac muscle, resulting in changes to cardiomyocytes, the coronary microvasculature, and the cardiac interstitium [42]. In the retina, uncontrolled primary hypertension can lead to hypertensive retinopathy, which is one of several markers of target-organ damage in hypertension as per the Joint National Committee on Prevention, Detection, Evaluation, and Treatment of High Blood Pressure [43].

Recently, Chua et al. quantified OCTA findings in 197 eyes in 118 adults with systemic hypertension treated with antihypertensive medication and found a change—albeit not statistically significant—in the superficial retinal capillary microvasculature associated with adverse cardiac remodeling, including increased left ventricular muscle mass, increased cardiac interstitial volume, and poorer global longitudinal cardiac muscle strain, concluding that reduced superficial capillary density may be associated with markers of cardiac damage [44].

Our analysis has several limitations that warrant discussion. First, the relatively small sample size limited the statistical power. Additionally, the small sample size limited our ability to assess the relationship between pre-existing poorly controlled hypertension and PEVAC development. Furthermore, the study was retrospective, and the data included were derived from a single tertiary medical center in which the patients underwent an SS-OCTA analysis on their first visit. We therefore were unable to determine the phase of the PEVAC lesions; we were also unable to determine whether the tight control of blood pressure was associated with the regression of the PEVAC lesions, or why some eyes developed a single lesion while others developed multifocal lesions. Furthermore, following the diagnosis of PEVAC, and due to both the reduction in visual acuity and the presence of intraretinal cystic spaces, one of the five eyes in our study was treated with anti-VEGF injections. To date, no prospective study has examined the potential role of anti-VEGF injections as a therapeutic option for PEVAC lesions. Thus, further studies are warranted in order to determine the effect of chronic systemic disease on the development of PEVAC lesions, particularly the putative effect of subtle changes in systemic blood resistance on the long-term progression of PEVAC.

Given that PEVAC is a localized problem and affects a specific area, it is possible that global vascular density parameters, which represent summations from the entire 6 × 6 mm scan, may not be significantly different between PEVAC and control groups. Since PEVAC primarily manifests as isolated, round hyperreflective walls surrounding vessel lumens in the perifoveal region, the impact on global vascular density measurements may be limited.

Based on our analysis, while focal changes were measured in the retina slab with respect to macular vascular density in the angio en face zone in PEVAC cases, there were no significant differences in macular vessel density between PEVAC cases and age-matched control eyes. This suggests that PEVAC may not be associated with a global reduction in macular vessel density but rather with more localized changes.

## 5. Conclusions

Despite its small sample size, this study provides valuable insights into the clinical and imaging characteristics of PEVAC. Most patients exhibited reduced visual acuity and metamorphopsia, but OCTA analysis of retinal vessel density revealed no significant differences compared to age-matched controls. These results suggest that PEVAC may not involve a global reduction in macular vessel density but rather more localized alterations. Additionally, the frequent presence of intraretinal cystic spaces and the associations between PEVAC and systemic factors such as hypertension and dyslipidemia underscore a complex relationship, potentially warranting reconsideration of PEVAC as an idiopathic condition.

This study highlights the need for further research on PEVAC, emphasizing the importance of thorough retinal evaluations and the influence of systemic health on retinal diseases. Expanding our understanding of these connections could guide future therapeutic strategies and enhance patient outcomes.

## Figures and Tables

**Table 1:** Demographic characteristics, clinical findings, and structural OCT findings of patients with PEVAC and subjects in the control group.

Patient	Age/Sex	Systemic Medical History	Eye with PEVAC (Number of Lesions)	Location of PEVAC Lesion(s)	Coincident Eye Disease
PEVAC Group					
P-1	85/Female	HTN, dyslipidemia	OS (1)	Inner temporal	OU: Glaucoma, OS: Pseudophakia
P-2	73/Female	HTN, hypothyroidism	OS (1)	Central	None
P-3	56/Male	HTN, dyslipidemia	OS (4)	Inner temporal	None
P-4	78/Male	Dyslipidemia, BPH	OS (1)	Inner temporal	None
P-5	58/Male	HTN, DM type 2, dyslipidemia	OD (1)	Inner inferior	None
**Control Group**					
C-1	60/Male	None	OD	None	None
C-2	59/Female	None	OD	None	None
C-3	63/Female	HTN, DM type 2	OD	None	None
C-4	65/Male	HTN, dyslipidemia	OS	None	None
C-5	77/Female	HTN	OS	None	None
C-6	63/Male	None	OD	None	None
C-7	78/Male	None	OD	Pseudophakia	None
C-8	71/Male	HTN	OD	Pseudophakia	None
C-9	63/Female	None	OS	None	None

PEVAC, perifoveal exudative vascular anomalous complex; HTN, systemic hypertension; DM type 2, non-insulin-dependent diabetes mellitus; BPH, benign prostatic hyperplasia; OU, both eyes; OD, right eye; OS, left eye.

**Table 2:** Clinical characteristics and OCT findings of 5 eyes in 5 patients with PEVAC at presentation (baseline) and at the follow-up visit.

Patient	Eye	Initial Presentation	Last Follow-Up	Follow-Up Period (Months)	Anti-VEGF Injections
P-1	OS	VA: 20/32, CMT: 286 μm, intraretinal cysts, no drusen	VA: 20/25, CMT: 270 μm, no intraretinal cysts, no drusen	14	10
P-2	OS	VA: 20/25, CMT: 271 μm, intraretinal cysts, no drusen	VA: 20/20, CMT: 259 μm, intraretinal cysts, no drusen	4	0
P-3	OS	VA: 20/25, CMT: 433 μm, intraretinal cysts, no drusen	VA: 20/25, CMT: 428 μm, intraretinal cysts, small reticular pseudo-drusen	4	0
P-4	OS	VA: 20/25, CMT: 282 μm, no intraretinal cysts, no drusen	VA: 20/25, CMT: 280 μm, intraretinal cysts, no drusen	13	0
P-5	OD	VA: 20/32, CMT: 335 μm, intraretinal cysts, no drusen	VA: 20/32, CMT: 335 μm, intraretinal cysts, no drusen	4	0

OCT, ocular coherence tomography; PEVAC, perifoveal exudative vascular anomalous complex; OD, right eye; OS, left eye.

## Data Availability

The original contributions presented in the study are included in the article/Supplementary Materials, further inquiries can be directed to the corresponding author.

## Supplementary Materials

The following supporting information can be downloaded at: https://www.mdpi.com/article/10.3390/jcm13226879/s1, Table S1: Summary of retinal angio en face macular density measured using ARI network analysis algorithms for PEVAC and control eyes. Table S2: Comparison of retinal angio en face macular vascular density measured using ARI network analysis algorithms between PEVAC and control eyes.
