# Supplementary material for: Perifoveal Exudative Vascular Anomalous Complex (PEVAC): Retinal Vascular Density Findings
Source: J Clin Med. 2024 Nov 15;13(22):6879. doi: 10.3390/jcm13226879 (PMC11595356; doi:10.3390/jcm13226879)
Supplement: Supplementary file 1 [file jcm-13-06879-s001.zip › jcm-3194556-supplementary.pdf]

Journal of Clinical Medicine

MDPI

Supplementary Materials

Perifoveal Exudative Vascular Anomalous Complex (PEVAC): Retinal Vascular Density Findings

Hamzah Aweidah [1], Deborah Cosette [2], Natan Lishinsky-Fischer [1], Tarek B. Eshak [3], Tomer Batash [1], Itay Chowers [1], Tareq Jaouni [1], Nadav Levinger [1] and Jaime Levy [1,*]

1 Department of Ophthalmology, Hadassah Medical Center, Faculty of Medicine, The Hebrew University of Jerusalem, Jerusalem 91120, Israel; hamzah.aweidah@mail.huji.ac.il (H.A.); natan.lishinsky@mail.huji.ac.il (N.L.-F.); batasht@gmail.com (T.B.); chowers@hadassah.org.il (I.C.); tareq@hadassah.org.il (T.J.); nadav.levinger@gmail.com (N.L.)
2 Carl Zeiss Meditec, Inc., Dublin, CA 94568, USA; deborah.cosette@zeiss.com
3 Department of Public Health Sciences, College of Health Professions, Slippery Rock University, Slippery Rock, PA 16057, USA; tarek.eshak@sru.edu
* Correspondence: levjaime@gmail.com

SUPPLEMENTARY MATERIALS

Supplementary Table S1. Summary of retinal angio en face macular density measured using ARI network analysis algorithms for PEVAC and control eyes.

Patient number (eye)	Retinal Angio En Face Macular Density in PEVAC Eyes (n=5)								
	Cen-tral	Inner zones				Outer zones			
		Nasal	Superior	Temporal	Inferior	Nasal	Superior	Temporal	Inferior
P-1 (OS)	6.36	16.35	15.88	12.62	16.46	19.86	17.93	12.16	16.80
P-2 (OS)	15.92	22.02	20.80	22.16	22.68	22.27	22.46	18.49	22.08
P-3 (OS)	10.33	19.10	18.21	19.07	17.98	19.12	19.61	19.15	19.87
P-4 (OS)	6.84	16.26	17.17	13.97	16.93	21.37	19.43	16.72	19.40
P-5 (OD)	11.29	21.71	21.02	21.31	20.46	22.23	18.78	17.29	20.89
Control subject number (eye)	Retinal Angio En Face Macular Density in Control Eyes (n=9)								
	Cen-tral	Inner zones				Outer zones			
		Nasal	Superior	Temporal	Inferior	Nasal	Superior	Temporal	Inferior
C-1 (OD)	15.30	21.41	20.48	22.37	21.44	23.43	21.20	20.85	21.64
C-2 (OD)	6.61	18.27	17.96	17.32	18.55	21.88	20.34	17.54	21.00
C-3 (OD)	14.19	22.06	22.08	21.36	21.66	22.62	20.74	20.78	21.27
C-4 (OS)	18.37	20.14	18.00	20.72	21.22	21.22	20.86	19.80	22.13
C-5 (OS)	7.03	14.01	17.00	18.89	15.63	20.96	19.78	18.70	18.18
C-6 (OD)	6.06	18.61	20.25	17.89	16.56	20.89	20.18	18.27	15.68
C-7 (OS)	9.89	21.89	20.98	17.26	20.52	22.62	20.87	16.74	17.66
C-8 (OD)	1.71	18.81	11.94	5.06	14.06	23.67	14.70	5.58	14.99
C-9 (OS)	7.57	21.50	21.96	20.34	18.76	22.60	20.98	18.24	17.52

OD, right eye; OS, left eye, FOV, fovea; PEVAC, perifoveal exudative vascular anomalous complex

Supplementary Table S2. Comparison of retinal angio en face macular vascular density measured using ARI network analysis algorithms between PEVAC and control eyes.

Retinal Zone	PEVAC Eyes (n=5)	Control Eyes (n=9)	p-value
Central	10.15 (3.87) [5.34-14.95]	9.64 (5.30) [5.56-13.71]	0.79
Inner Zone			
Nasal	19.09 (2.78) [15.63-22.54]	19.63 (2.58) [17.65-21.61]	0.90
Superior	18.62 (2.25) [15.82-21.41]	18.96 (3.20) [16.50-21.42]	0.80
Temporal	17.83 (4.32) [12.47-23.18]	17.91 (5.15) [13.95-21.87]	0.99
Inferior	18.90 (2.62) [15.65-22.15]	18.71 (2.77) [16.58-20.84]	0.99
Outer Zone			
Nasal	20.97 (1.42) [19.20-22.74]	22.21 (1.03) [21.42-23.00]	0.14
Superior	19.64 (1.71) [17.52-21.76]	19.96 (2.02) [18.41-21.52]	0.24
Temporal	16.76 (2.75) [13.35-20.17]	17.39 (4.64) [13.82-20.96]	0.36
Inferior	19.81 (1.97) [17.36-22.26]	18.90 (2.68) [16.83-20.96]	0.80
